# Irreversible Anterior Ischemic Optic Neuropathy Complicating Interferon Alpha and Ribaverin Therapy

**DOI:** 10.4061/2011/814242

**Published:** 2010-12-19

**Authors:** Hassan Seddik, Mouna Tamzaourte, Fadoua Rouibaa, Maha Fadlouallah, Ahmed Benkirane

**Affiliations:** ^1^Hepatogastroenterology 2 Unit, Mohamed V Military Hospital, Rabat, Morocco; ^2^19, Rue Oued El Makhazine No. 7, Agdal, Rabat, Morocco; ^3^Hepatogastroenterology 1 Unit, Mohamed V Military Hospital, Rabat, Morocco

## Abstract

Ophthalmologic complications with interferon therapy are rare and usually reversible. The anterior ischemic optic neuropathy is an uncommon complication of interferon treatment. A case of irreversible anterior ischemic optic neuropathy complicating interferon therapy for chronic hepatitis C is reported. We suggest that periodic ophthalmological examinations, including visual acuity and fundus examinations, should be performed to patients with high risk of ocular complications after starting and during treatment. We also suggest that an ophthalmologist would be able to detect these complications. Antiviral treatment should be stopped immediately if severe ophthalmologic complications occur.

Ophthalmologic complications with interferon therapy are rare, usually mild and reversible, and do not require the withdrawal of antiviral treatment [1]. Ophthalmologic complications with interferon alpha therapy, such as retinopathy with cotton-wool spots, hemorrhages and macular edema, optic neuropathy, and thrombotic microangiopathy occur in less than 1% of treated patients. Individuals with diabetes, hypertension, dislipidemia, and hypercoagulable states are more prone to develop those changes. In most cases, they are subclinical, mild, and reversible, not requiring the withdrawal of the treatment [2]. We report a case of a patient with chronic hepatitis C who developed irreversible anterior ischemic optic neuropathy during antiviral treatment by pegylated interferon combined with ribavirin.

A 55-year-old man had chronic hepatitis C since 2008. HCV genotype was 4, HCV-RNA level was 6,08·10^6^ equivalent/ml and the histological score of the liver biopsy was A2 F3 according to the METAVIR classification. Pegylated interferon (180 *μ*g/ week) combined with ribavirin (1000 mg/day) was started in June 2008. HCV-RNA had been undetectable since week 12 of the therapy. Five months after starting treatment, the patient presented with a sudden bilateral decreased vision. Visual acuity was 2/10 in the left eye and 4/10 in the right eye, while his visual acuity was previously normal. Fundus examination revealed bilateral disc edema. Fluorescein angiography confirmed the bilateral anterior ischemic optic neuropathy. There was no response to the visual evoked potentials in the either eye. The complete blood count chemistry, sedimentation rate, C-reactive protein level, antinuclear antibodies, anti-DNA antibodies, and pANCA antibodies were normal. Cryoglobulinemia was negative as were magnetic resonance imaging of the head, echocardiogram, and carotid Doppler study. Antiviral treatment was discontinued, and the patient received methyl prednisolone 1 g for 5 days. An ophthalmologic examination one month later showed that visual acuity had not clearly improved the visual acuity was 3/10 for the left eye and 5/10 for the right one, while optic disc oedema was partially resolved. HCV- RNA was always undetectable six months and 1 year after interruption of treatment, suggesting a sustained virological response. Ophthalmologic examination after 1 years showed a visual acuity of 3/10 for the left eye and 5/10 for the right one.

Anterior ischemic optic neuropathy is an uncommon complication of interferon treatment which may dramatically impair visual function. Few cases have been reported in the literature [3] during antiviral treatment for chronic hepatitis C [2] and also during interferon treatment of cancer (2 cases), treatment of primary thrombocytemia (1 case), treatment of malignant melanoma (1 case), and during treatment of amyotrophic lateral sclerosis [4]. Predisposing factors have not been clearly identified, except for classic vascular risk factors (diabetes, arterial hypertension, and dyslipidemia) [2]. In our patient's case, no vascular risk factors were found. Purvin and Col [5] has suggested involvement of the posterior ciliary arterius rather than the retinal vessels as a possible cause of anterior ischemic optic neuropathy [5]. Lohmann and Coll hypothesized that interferon could produce autoantibodies, and thus cause desposition of immune complexes in the small retinal or optic cytokines causing an inflammatory reaction of the blood vessels that could then lead to ischemia [6]. The favorable course is uncommon in anterior ischemic optic neuropathy with vascular causes suggesting another mechanism [4]. The absence of resolution of anterior ischemic optic neuropathy after discontinuation of interferon in our patient suggests probable vascular causes. In the literature, the course of anterior ischemic optic neuropathy occurring during interferon treatment for chronic hepatitis C was favorable in four cases [7], and not resolved in three cases [2, 4]. After interruption of the antiviral treatment, our patient received methyl prednisolone, a steroid drug prescribed by the ophthalmologists, without favorable course of visual function. In the literature, two patients received steroids after interferon treatment was discontinued, but there was no improvement in visual function [3]. 

Anterior ischemic optic neuropathy is uncommon unpredictable, and the frequency is unknown with interferon therapy. Periodic ophthalmologic examinations before starting treatment and during treatment particularly in patients with vascular risk factors must be indicated. Therapy should be stopped immediately if severe ophthalmologic complications occur. 
